# The Beat

**DOI:** 10.1289/ehp.120-a389

**Published:** 2012-10-01

**Authors:** Erin E. Dooley

## Worms Reduce Metal Load in Organic Waste

Vermicomposting uses worms to break down organic waste into rich material that can be used as fertilizer. An Indian study adds to growing evidence that this process may help remove toxic heavy metals such as lead and cadmium from urban organic waste.^1^ Tests of vermicompost produced from municipal solid waste, market vegetable waste, and floral waste showed significantly lowered metal content compared with the raw waste. Of the three worm species assessed, *Eudrilus eugeniae* appeared the most efficient at removing metals. Earlier work by these authors indicated that worms’ body burden of metals was inversely associated with the metal content of the vermicompost they produced.^2^

**Figure:**
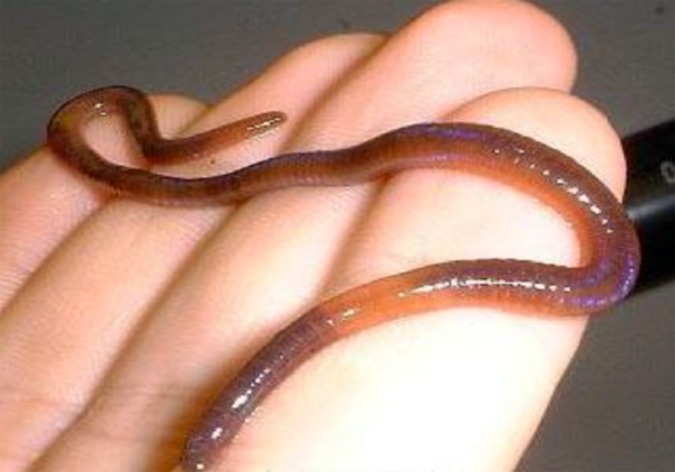
Worms may offer a way to turn contaminated waste into clean fertilizer. © M. Vikram Reddy

## Low-Level Lead and Gout

Gout is a form of arthritis caused by a buildup of uric acid in the blood. High lead exposures have long been associated with gout in adults. Now a new analysis of NHANES data examines potential associations with low-level lead exposure like that found in the general population. The study authors report a 3.6-times higher prevalence of gout and 1.9-times higher prevalence of elevated blood levels of uric acid among people with the highest blood lead levels (mean 3.95 µg/dL), compared with those with the lowest blood lead levels (mean 0.89 µg/dL).^3^ The link remained after adjusting for factors including history of diabetes, income, and smoking.

## Initiative to Reduce Emissions from Traditional Brick Making

In September 2012 the Climate and Clean Air Coalition to Reduce Short-Lived Climate Pollutants kicked off a new initiative to modernize traditional brick making in developing countries. More efficient production can reduce emissions of black carbon and other pollutants by 10–50%, depending on the process, scale, and fuel used. The first phase of the initiative began with a three-day capacity-building workshop in Guanajuato, Mexico, where participants shared their knowledge about existing cleaner alternatives to traditional brick making. By the end of 2013 the coalition plans to have profiled current brick production for each participating region.^4^

**Figure:**
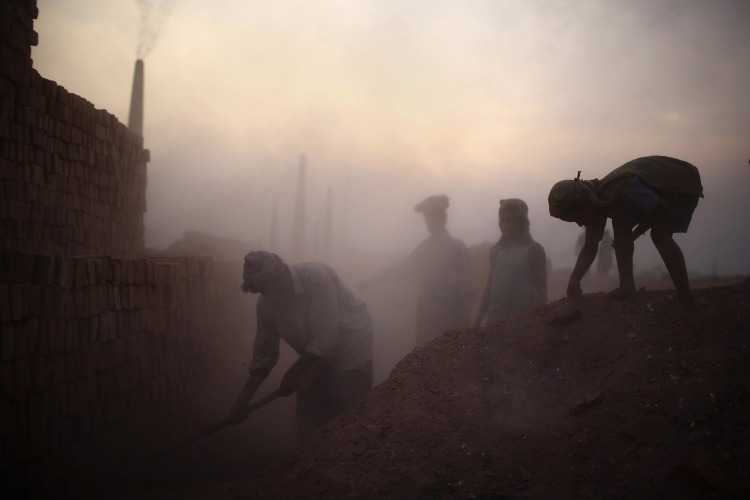
Traditional brick making produced 5.5% of the world’s black carbon emissions in 2010.^8^ © G.M.B. Akash/Panos Pictures

## Potential New Biosensor for Arsenic in Drinking Water

High levels of naturally occurring arsenic are found in drinking water around the world. Most current methods for detecting arsenic in water involve a long analysis time. A proposed new biosensor combines an artificial single-stranded oligonucleotide sequence (“aptamer”) specifically designed to bind arsenic, gold nanoparticles (which respond to arsenic by changing color), and a cationic surfactant that causes the gold nanoparticles to aggregate in the presence of arsenic.^5^ In under 2 minutes, investigators could visually detect arsenic concentrations as low as 40 ppb, and concentrations as low as 0.6 ppb were detectable using colorimetric and resonance scattering assays. The World Health Organization has set a provisional guideline of 10 µg/L (10 ppb) for arsenic in drinking water.^6^

**Figure:**
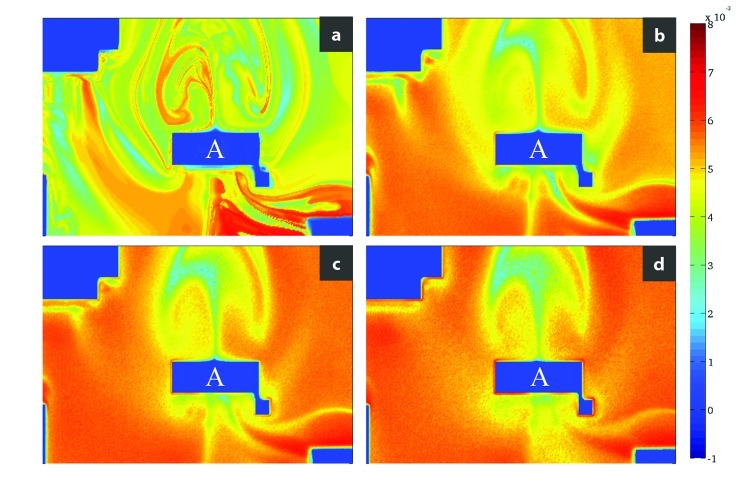
Predominantly northern-bound urban flow near a tall building (“A”). Frame (a) depicts deterministic (i.e., nonvarying) flow, while frames (b) through (d) introduce random variables. W. Tang et al., doi:10.1063/1.4729453

## Model Simulates Flow Patterns of Urban PM

Pollutants are carried through city air in patterns known as urban flows. Urban flows are influenced by a number of factors including building density, presence of green spaces, and traffic. Using a new mathematical model, researchers simulate how urban flows influence the creation of “inertial Lagrangian coherent structures”of particulate matter (PM)—essentially, sustained vortices that may expose people to high densities of particles.^7^ The researchers applied their model to the Hermoso Park region of Phoenix, Arizona, where PM has been blamed for high levels of respiratory illness. The model may be useful for advising policy makers and public health officials of areas with high pollutant loads.
